# The association of body mass index with functional dyspepsia is independent of psychological morbidity: A cross-sectional study

**DOI:** 10.1371/journal.pone.0245511

**Published:** 2021-01-26

**Authors:** Keng Hau Beh, Kee Huat Chuah, Nurul Azmi Mahamad Rappek, Sanjiv Mahadeva

**Affiliations:** 1 Department of Medicine, University of Malaya, Kuala Lumpur, Malaysia; 2 Gastroenterology and Hepatology Unit, Department of Medicine, University of Malaya, Kuala Lumpur, Malaysia; 3 Staff and Student Health Clinic, University Malaya Medical Centre, Kuala Lumpur, Malaysia; Hong Kong Polytechnic University, HONG KONG

## Abstract

**Background and aim:**

The association between body mass index (BMI) and functional gastrointestinal disorders (FGIDs) has been inconsistent. We aimed to explore the association of BMI with FGIDs in a primary care setting to provide more data in this area.

**Methods:**

A cross-sectional study of consecutive Asian adults attending a primary healthcare setting was conducted. This study was conducted in 2 phases: The association between BMI and common FGIDs (functional diarrhea/FD, irritable bowel syndrome/IBS, functional diarrhea and functional constipation/FC) was studied initially. The influence of anxiety and depression on BMI and FGIDs was additionally explored in phase 2.

**Results:**

A total of 1002 subjects (median age 32 years, 65.4% females, 90.7% Malay ethnicity, 73.2% higher than secondary level education) were recruited between August 2019 to January 2020. The majority of subjects were obese (39.2%), and had central obesity (51.7%), while 6.1% had metabolic syndrome. The prevalence of FD, IBS, functional diarrhea and FC were 7.5% (n = 75), 4.0% (n = 40), 1.2% (n = 12) and 10.5% (n = 105) respectively, based on the Rome III criteria. Among individual FGIDs, FD subjects had more underweight adults (BMI<18.5kg/m^2^) compared to controls (13.3% vs 3.5%, P = 0.002) and being underweight remained as an independent association with FD [OR = 3.648 (95%CI 1.494–8.905), P = 0.004] at multi-variate analysis. There were no independent associations between BMI and other FGIDs. When psychological morbidity was additionally explored, anxiety (OR 2.032; 95%CI = 1.034–3.991, p = 0.040), but not depression, and a BMI<18.5kg/m^2^ (OR 3.231; 95%CI = 1.066–9.796, p = 0.038) were found to be independently associated with FD.

**Conclusions:**

FD, but not other FGIDs, is associated with being underweight. This association is independent of the presence of anxiety.

## Introduction

Functional gastrointestinal disorders (FGIDs) are disorders with recurrent and chronic gastrointestinal (GI) symptoms in the absence of structural or biochemical abnormalities that can be identified by routine investigations. At present, symptom-based criteria, developed by the Rome Foundation have been accepted as the gold standard for diagnosing FGIDs [1–4]. FGIDs are common and an estimated 40% of the world’s population are suffering from this condition [5]. Although FGIDs do not cause mortality, the quality of life and productivity of patients with FGIDs are consistently reported to be significantly affected, in addition to increasing the healthcare burden of individual countries [6–8].

The epidemiological associations of FGIDs have been known to include gender, age, race, genetics, geographic area and food [9]. The association of BMI with FGIDs has been explored in a few studies, but the available data is conflicting. Le pluart et al reported that a greater association with FD was observed for underweight and obesity, while the risk of functional diarrhea was increased with BMI among females [10]. On the contrary, a cross-sectional study of 4,763 Iranian adults by Akhondi et al. showed no association between BMI and IBS [11]. Meanwhile, a previous review article which evaluated 11 studies has suggested that the frequency of IBS and obesity was variable [12].

With the growing epidemic of obesity in the Asia-Pacific region, determining the association between BMI and FGIDs could enhance the understanding of the pathophysiology of FGIDs and potentially provide a simple method of treating a complex group of conditions.

Functional dyspepsia (FD), irritable bowel syndrome (IBS), functional diarrhea, and functional constipation (FC) are among the most established FGIDs. We aimed to explore the association between BMI and FGIDs (FD, IBS, functional diarrhea and FC) in an effort to provide more data in this area of FGID epidemiology. The influence of anxiety and depression (well known to co-exist with FGIDs) on BMI and FGIDs was additionally explored.

## Methods

### Study design and participants

A questionnaire-based, cross sectional study of consecutive adults (>18 years old) who attended a staff health facility was conducted in this large, teaching institution from August 2019 to January 2020. In the context of the Malaysian Healthcare Service, the staff health clinic usually serves as a primary care clinic for staff of both the hospital and the University of Malaya, the staffs’ dependents/ immediate relatives and students of health and allied-care programs. We excluded the following from participation: subjects who were pregnant, had diabetes mellitus, previous gastrointestinal surgery (except appendicectomy) or confirmed organic gastrointestinal disease, including peptic ulcer disease, gastrointestinal malignancy, inflammatory bowel disease and coeliac disease were excluded. Subjects with warning symptoms (eg. dysphagia, unexplained anemia, melena, hematemesis, rectal bleeding or significant unintentional weight loss for past 6 months) but without endoscopy or imaging (to rule out organic disease) were additionally excluded. The study conformed to the ethical guidelines of the 1975 Declaration of Helsinki and ethical approval was obtained from the University Malaya Medical Centre Medical Research Ethics Committee prior to commencement (Reference No.: 2019727–7692, 20 August 2019).

### Procedures

Face-to-face interviews were conducted by a single investigator (KHB), using a self-designed questionnaire based on the translated, validated Asian Rome III questionnaire. A standardized approach with reference to a simple diagram of the abdomen (for location of site of symptoms) and the Bristol stool chart were applied [13]. A face-to-face interview method was used as we anticipated a lower education level amongst our participants. Adults who were able to provide written consent were recruited. This study was conducted in 2 phases: i) the 1^st^ phase included assessment of parameters without psychological disease assessment, mainly to study the BMI associations with FGID; ii) the 2^nd^ phase involved a sub-group assessment for psychological disorders from the original cohort. The questionnaire consisted of the following information: basic socio-demographic information (age, gender, ethnicity, educational level, monthly income), lifestyle data (physical activity, smoking, alcohol consumption), comorbidities, specific gastrointestinal symptoms. Monthly income of less than USD 750, USD 750 to 1249 and more than USD 1250 were considered as low, middle and high income respectively. Physical activity level was categorized into low, moderate and high level based on the International Physical Activity Questionnaire Short Form (IPAQ-SF) [14]. The diagnosis of FD, IBS, functional diarrhea and FC were based on the Rome III [2,3], instead of the latest Rome IV diagnostic criteria, due to the lower detection of IBS in the latter [5,15,16]. A recent systemic review concluded that the prevalence of IBS was substantially lower with the Rome IV criteria and suggested that the restrictive criteria might be less suitable than Rome III for large epidemiological studies [17]. Subjects who did not fulfil the diagnostic criteria for FD, IBS, functional diarrhea and FC were used as controls.

Anthropometric measurements of recruited subjects were also obtained using a standard protocol. Underweight, normal weight, overweight and obesity were defined as body mass index (BMI) <18.5kg/m^2^, 18.5 to 22.9kg/m^2^, 23.0 to 27.4kg/m^2^, ≥27.5kg/m^2^ respectively. Based on body fat equivalence and comorbid disease risk, BMI of 23 and 27 have been recommended as the cut-off points for public health action according to Malaysia and Singapore Clinical Practice Guidelines [18,19]. Central obesity was defined as waist circumference >90 cm for men and >80 cm for women [20].

In the 2^nd^ phase, a consecutive sub-group of subjects were further assessed for anxiety and depression by a validated English and Malay version of hospital anxiety and depression scale (HADS) consisting of 14 questions with seven items for each subscale of anxiety or depression. The total score for each subscale of depression or anxiety ranges from 0 to 21. Significant anxiety or depression was defined as a score of ≥8 for each respective scale [21,22].

### Sample size calculation

Based on an estimated 7% difference in GI symptoms between normal and obese weight patients [23], we calculated that a minimum of 405 subjects (control) and 203 subjects (FGIDs) at 2:1 ratio in two groups will be required to achieve a 90% statistical power at the 0.05 significance level.

### Statistical analysis

All data were analyzed with IBM SPSS Statistics software (version 25). Continuous variables were expressed as median (interquartile range) and compared across different groups using Mann-Whitney U test. Categorical variables were expressed as percentage and evaluated using Pearson chi-squared or Fisher’s exact test, as appropriate. BMI associations with FGIDs were additionally analyzed with logistic regression using either normal weight or overweight as reference. Variables with p-value of ≤0.3 and clinical relevance were then entered into a multivariate logistic regression to identify independent risk factors of FGIDs. Results were expressed as odds ratio with 95% confidence interval. In all tests, a p-value of less than 0.05 was considered statistically significant.

## Results

**Fig 1:**
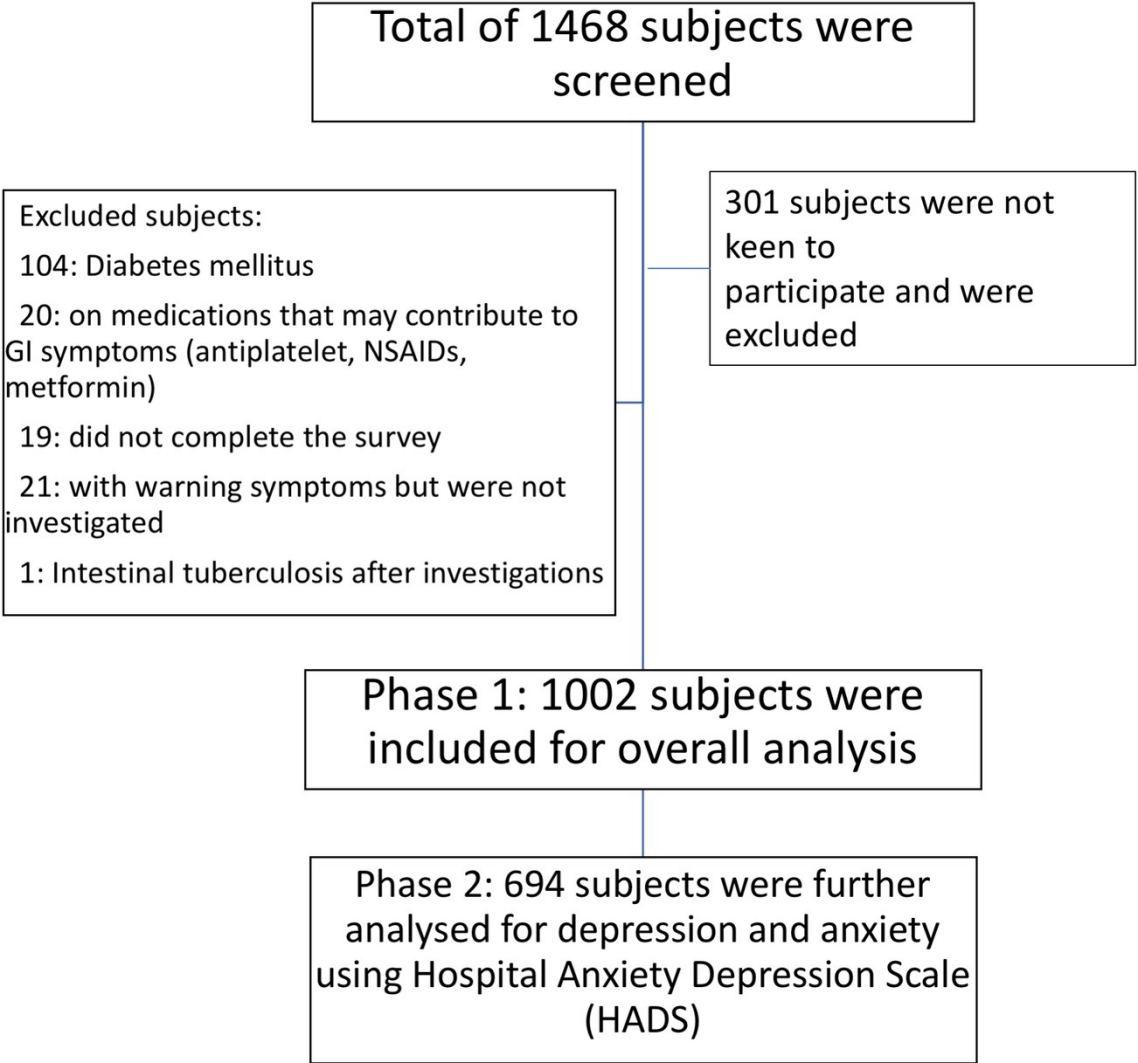
Flow chart of study population.

A total of 1002 subjects (68%) were recruited **(Fig 1)** between August 2019 to January 2020 in the 1^st^ phase of the study.

The median age of the study population was 32 years, 65% (n = 655) were female, and the majority of subjects were of Malay ethnicity (91%, n = 909). The majority of subjects were obese (39.2%, n = 392) and had central obesity (51.7%, n = 518), while 6.1% (n = 61) had metabolic syndrome.

In terms of habits, most of the subjects were non-smokers (83%, n = 833) and teetotalers (94%, n = 940). Four hundred and forty eight (44.7%) had a sedentary lifestyle, i.e. they had low levels of physical activity. Socio-economically, 52.5% (n = 526) of subjects had vocational/ college education level (52.5%, n = 526) and were from a low income group (38.1%, n = 382) **(Table 1)**.

**Table 1:** Characteristics of study subjects.

	Overall		Non-FGID		FGIDs		P Value
	(N = 1002)		(N = 795)		(N = 207)		
	n	(%)	n	(%)	n	(%)	
Median age	32 (26–40)		32 (26–40)		31 (25–38)		0.066
Female	655	(65.4)	513	(64.5)	142	(68.6)	0.273
Body mass index							
Less than 18.5	41	(4.1)	28	(3.5)	13	(6.3)	0.123
18.5–22.9	241	(24.1)	191	(24.0)	50	(24.2)	Reference
23.0–27.4	327	(32.6)	257	(32.3)	70	(33.8)	0.849
27.5 and above	393	(39.2)	319	(40.1)	74	(35.7)	0.555
Central obesity	518	(51.7)	415	(52.2)	103	(49.8)	0.531
Metabolic syndrome	61	(6.1)	54	(6.8)	7	(3.4)	0.068
Ethnicity							0.274
Malay	909	(90.7)	723	(90.9)	186	(89.9)	
Indian	47	(4.7)	33	(4.2)	14	(6.8)	
Chinese	43	(4.3)	37	(4.7)	6	(2.9)	
Others	3	(0.3)	2	(0.3)	1	(0.5)	
Educational level							0.884
Never schooled	2	(0.2)	2	(0.3)	0	(0.0)	
Primary	10	(1.0)	8	(1.0)	2	(1.0)	
Secondary	257	(25.6)	205	(25.8)	52	(25.1)	
Vocational/college	526	(52.5)	420	(52.8)	106	(51.2)	
Tertiary	207	(20.7)	160	(20.1)	47	(22.7)	
Monthly income							0.745
Less than USD 750	531	(53.0)	418	(52.6)	113	(54.6)	
USD 750–1249	371	(37.0)	299	(37.6)	72	(34.8)	
USD 1250 or above	100	(10.0)	78	(9.8)	22	(10.6)	
Smoking status							0.293
Never smoke	833	(83.1)	658	(82.8)	175	(84.5)	
Former smoker	99	(9.9)	84	(10.6)	15	(7.2)	
Current smoker	70	(7.0)	53	(6.7)	17	(8.2)	
Drinking status							0.620
Lifetime abstainer	940	(93.8)	745	(93.7)	195	(94.2)	
Ex-drinker	23	(2.3)	20	(2.5)	3	(1.4)	
Current drinker	39	(3.9)	30	(3.8)	9	(4.3)	
Level of physical activity							0.488
Low	448	(44.7)	365	(45.9)	83	(40.1)	
Moderate	319	(31.8)	247	(31.1)	72	(34.8)	
High	139	(13.9)	107	(13.5)	32	(15.5)	
Unknown	96	(9.6)	76	(9.6)	20	(9.7)	

FGID, Functional gastrointestinal disorder; USD, US Dollar.

**Fig 2:**
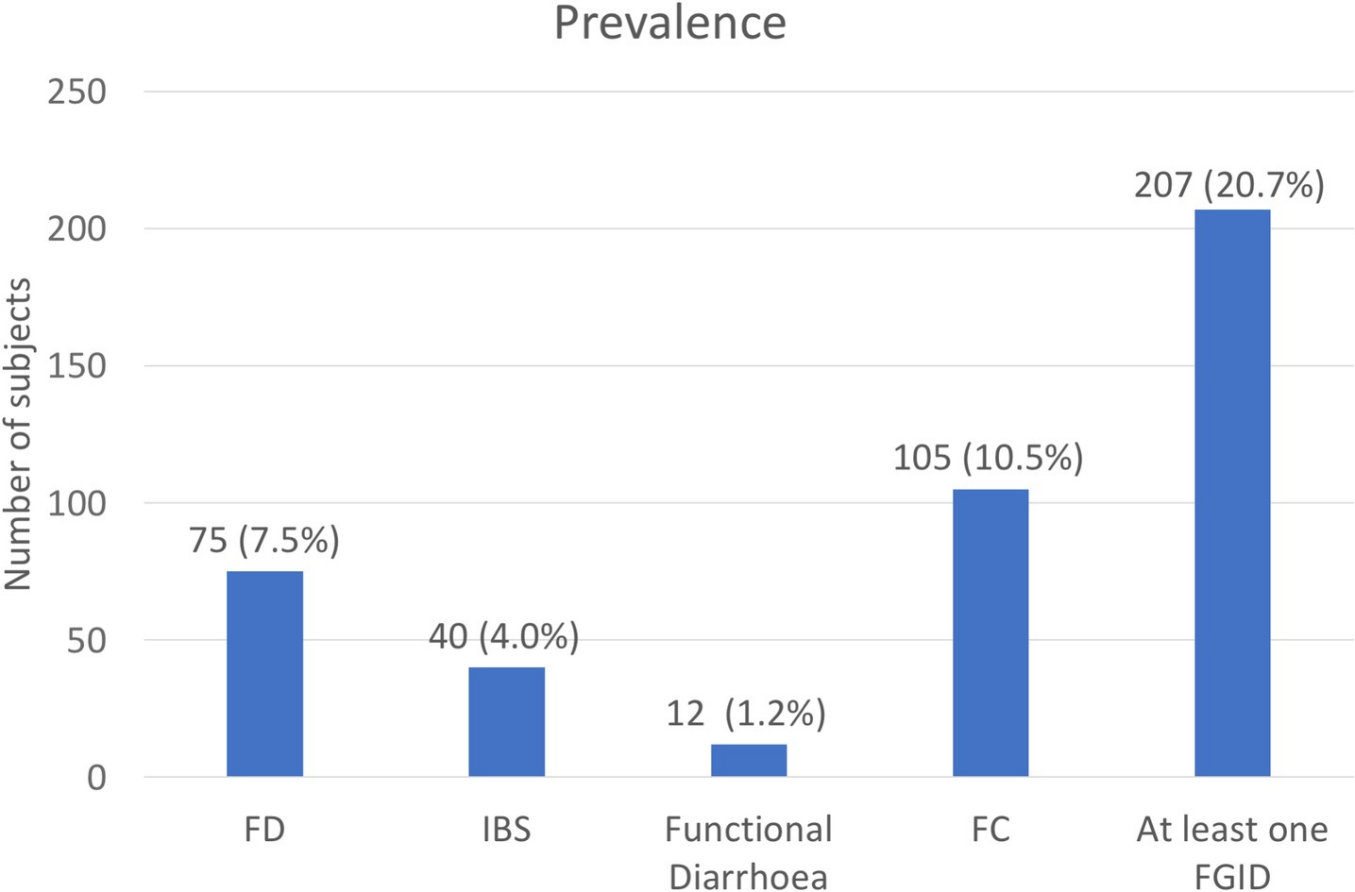
Prevalence of FGIDs.

The prevalence of FD, IBS, functional diarrhea and FC amongst the study population was 7.5% (n = 75), 4.0% (n = 40), 1.2% (n = 12) and 10.5% (n = 105) respectively **(Fig 2)**.

### BMI association with FGIDs

The prevalence of underweight and obesity among subjects with FGIDs vs non-FGIDs controls were similar (underweight: 6.3%, n = 13 vs 3.5%, n = 28, P = 0.123; obesity: 35.7%, n = 74 vs 40.1%, n = 319, P = 0.555). Neither central obesity nor metabolic syndrome were found to be associated with FGIDs (central obesity: 49.8%, n = 103 in FGIDs vs 52.2%, n = 415 in controls, P = 0.531; metabolic syndrome: 3.4%, n = 7 in FGIDs vs 6.8%, n = 54 in controls, P = 0.068) **(Table 1)**.

Among individual FGIDs, FD subjects had a higher proportion of underweight adults compared to controls (13.3%, n = 10 vs 3.5%, n = 28, P = 0.002) at univariate analysis. Otherwise, obesity rates were similarly distributed among FD and non-FGIDs (33.3%, n = 25 vs 40.1%, n = 319, P = 0.698) **(S1 Table)**. On multivariate analysis, being underweight (BMI < 18.5) remained as an independent association with FD [AOR = 3.648 (95% CI 1.494–8.905), P = 0.004], together with Indian ethnicity **(Table 2)**.

**Table 2:** Multivariate analysis of risk factors for functional dyspepsia (N = 870).

	OR	95% CI	P	AOR	95% CI	P
Age	0.989	0.967–1.011	0.324	1.005	0.981–1.030	0.667
Body mass index						
Less than 18.5	4.013	1.671–9.636	0.002	3.648	1.494–8.905	0.004
18.5–22.9	1	reference		1	reference	
23.0–27.4	1.005	0.523–1.934	0.987	0.976	0.478–1.993	0.946
27.5 and above	0.881	0.463–1.673	0.698	0.959	0.389–2.365	0.927
Central Obesity	0.759	0.472–1.222	0.256	0.970	0.473–1.990	0.935
Metabolic syndrome	0.185	0.025–1.360	0.097	0.202	0.026–1.556	0.125
Ethnicity						
Malay	1	reference		1	reference	
Indian	2.697	1.196–6.080	0.017	2.725	1.167–6.363	0.020
Chinese	0.601	0.142–2.551	0.490	0.609	0.137–2.705	0.515
Income						
Less than USD750	1			1	reference	
USD 750–1249	0.760	0.457–1.264	0.290	0.771	0.455–1.306	0.334
USD 1250 and above	0.466	0.163–1.332	0.154	0.509	0.171–1.515	0.225

OR, Odd ratio; CI, Confidence interval; AOR, Adjusted odd ratio.

Functional diarrhea subjects demonstrated a trend towards an association with obesity, but this did not achieve statistical significance (75%, n = 9 vs 40.1%, n = 319, P = 0.101). On the contrary, there was no significant association found between BMI and other FGIDs (IBS, underweight: 5.0%, n = 2, obesity: 35%, n = 14; FC, underweight: 1.9%, n = 2, obesity: 36.2%, n = 38) **(S1 Table)**.

Central obesity was found to be more common in subjects with functional diarrhea compared to controls (91.7%, n = 11 vs 52.2%, n = 415, P = 0.007) at univariate analysis **(S1 Table)**. Multivariate analysis was not performed to look for factors associated with functional diarrhea due to the small sample size of subjects with functional diarrhea. Otherwise, there did not appear to be any significant association with central obesity and other FGIDs.

### Influence of anxiety and depression on BMI association with FGIDs

A total of 694 subjects from the original cohort were assessed for anxiety and depression by HADS in the 2^nd^ phase of the study from October 2019 to January 2020. The demographics of this sub-group of adults compared to the 1^st^ phase of the study were similar **(S2 Table)**. The prevalence of FGIDs between the 2 phases were as follows: FD = 7.5% (phase 1) vs 7.6% (phase 2), IBS = 4.0% (phase 1) vs 3.9% (phase 2), functional diarrhea = 1.2% (phase 1) vs 1.3% (phase 2) and FC = 10.5% (phase 1) vs 9.7% (phase 2).

The prevalence of anxiety and depression in the 2^nd^ phase of study were 22.9% (n = 159) and 13.8% (n = 96) respectively.

Anxiety was more prevalent among individuals with FGIDs compared to controls (29.6%, n = 42 vs 21.2%, n = 117, P = 0.043) on univariate analysis. However, this association became not significant after adjustment for potential confounders [AOR of 1.494 (95% CI 0.983–2.269), P = 0.060] **(S3 Table)**

Among individual FGIDs, anxiety was found to be associated with FD (39.6%, n = 21 vs 21.2%, n = 117, P = 0.002) on univariate analysis. On multivariate analysis, only anxiety and BMI less than 18.5kg/m^2^ were consistently associated with FD with AOR of 2.032 (95% CI 1.034–3.991, P = 0.040) and 3.231 (CI 95% 1.066–9.796, P = 0.038) **(Table 3)**.

**Table 3:** Univariate and multivariate analysis of risk factors for FD with psychological disorders (N = 605).

	Non-FGID	FD		
	N = 552	N = 53		
			Univariate/ multivariate analysis	
	n (%)	n (%)	OR (95% CI)	AOR (95% CI)
Psychological Disorders				
Anxiety	117 (21.2)	21 (39.6) P = 0.005	2.440 (1.356–4.389) P = 0.003	2.032 (1.034–3.991) P = 0.040
Depression	73 (13.2)	12 (22.6) P = 0.065	1.920 (0.964–3.824) P = 0.063	1.248 (0.574–2.714) P = 0.576
Median age	32 (25–41)	29 (22–41) P = 0.202	0.987 (0.962–1.013) P = 0.339	0.994 (0.965–1.023) P = 0.661
Female	364 (65.9)	37 (69.8) P = 0.649		
Body mass index				
Less than 18.5	19 (3.4)	6 (11.3)	3.395 (1.139–10.116) P = 0.028	3.231 (1.066–9.796) P = 0.038
18.5–22.9	129 (23.4)	12 (22.6)	1	1
23.0–27.4	190 (34.4)	15 (28.3)	0.849 (0.385–1.872) P = 0.684	0.924 (0.407–2.097) P = 0.850
27.5 and above	214 (38.8)	20 (37.7) P = 0.050	1.005 (0.475–2.123) P = 0.990	1.060 (0.472–2.381) P = 0.887
Central obesity	282 (51.1)	24 (45.3) P = 0.473		
Metabolic syndrome	36 (6.5)	0 P = 0.063		
Ethnicity				
Malay	505 (91.5)	47 (88.7)	1	1
Chinese	29 (5.3)	2 (3.8)	0.741 (1.710–3.203) P = 0.688	0.613 (0.132–2.834) P = 0.531
Indian	16 (2.9)	4 (7.5)	2.686 (0.863–8.363) P = 0.088	2.042 (0.620–6.723) P = 0.240
Others	2 (0.4)	0 P = 0.308		
Educational level				
Never schooled	0			
Primary	5 (0.9)	1 (1.9)		
Secondary	135 (24.5)	14 (26.4)		
Vocational/ college	304 (55.1)	27 (50.9)		
Tertiary	108 (19.6)	11 (20.8) P = 0.866		
Monthly income				
Less than USD 750	300 (54.3)	32 (60.4)		
USD 750–1249	198 (35.9)	19 (35.8)		
USD 1250 or above	54 (9.8)	2 (3.8) P = 0.332		
Smoking status				
Never smoke	446 (84.4)	44 (83.0)		
Former smoker	51 (9.2)	7 (13.2)		
Current smoker	35 (6.3)	2 (3.8) P = 0.515		
Drinking status				
Lifetime abstainer	515 (93.3)	52 (98.1)		
Ex-drinker	16 (2.9)	0		
Current drinker	21 (3.8)	1 (1.9) P = 0.342		
Level of physical activity				
Low	250 (45.3)	24 (45.3)		
Moderate	165 (29.9)	17 (32.1)		
High	81 (14.7)	7 (13.2)		
Unknown	56 (10.1)	5 (9.4) P = 0.982		

FGID, Functional gastrointestinal disorder; FD, Functional dyspepsia; OR, Odd ratio; CI, Confidence interval; AOR, Adjusted odd ratio.

Anxiety was also found to be more common in subjects with FD-IBS overlap compared to control (71.4%, n = 5 vs 21.2%, n = 117, P = 0.007). However, multivariate analysis was not performed due to the small sample size of subjects with FD-IBS overlap.

**Fig 3:**
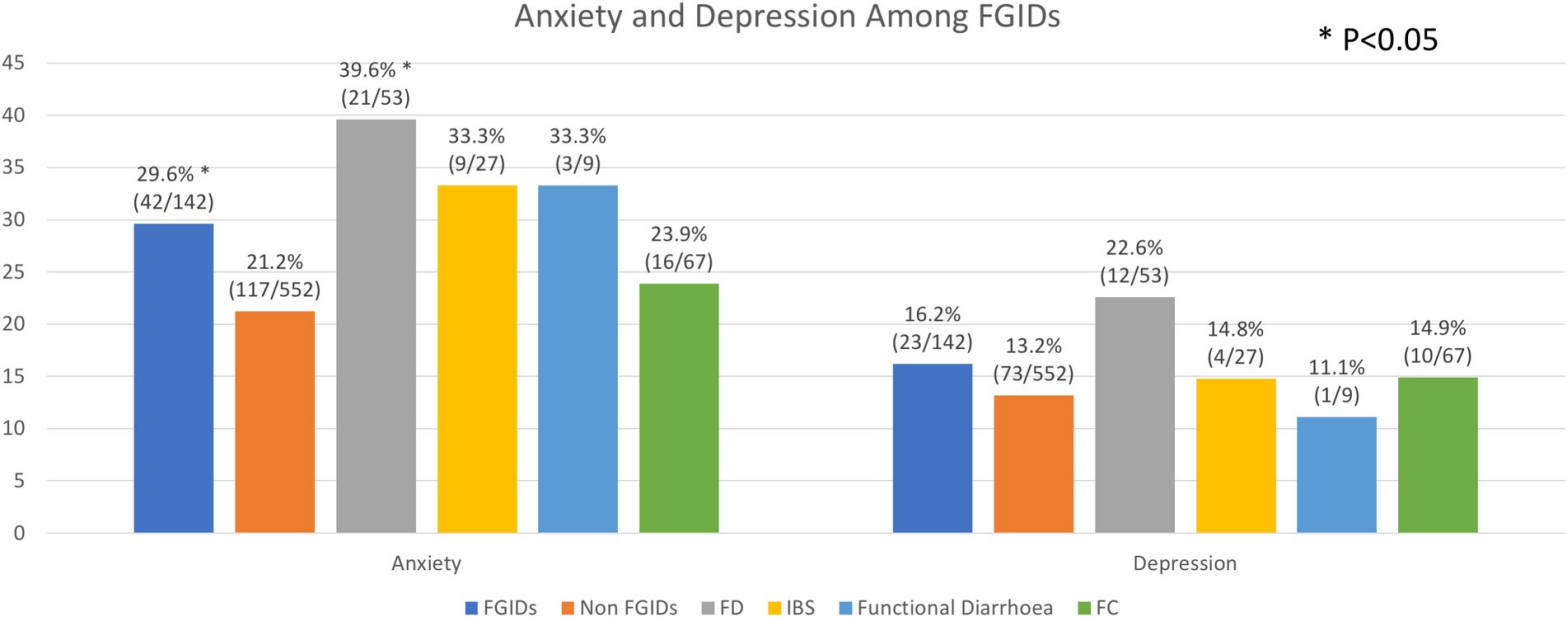
Anxiety and depression among FGIDs.

Subjects with IBS and functional diarrhea additionally demonstrated a trend towards an association with anxiety compared to non-FGIDs subjects (33.3%, n = 9, P = 0.152 in IBS; 33.3%, n = 3, P = 0.411 in functional diarrhea vs 21.2%, n = 117 in controls). There was a trend between depression and subjects with FGIDs, FD and FD-IBS overlap (16.2%, n = 23, P = 0.344 in FGIDs; 22.6%, n = 12, P = 0.065 in FD; 28.5%, n = 2, P = 0.239 in FD-IBS overlap vs 13.2%, n = 73 in controls). However this did not achieve statistical significance **(Fig 3)**.

## Discussion

This large cross-sectional study amongst adults attending primary care has shown that the prevalence of common FGIDs based on the Rome III criteria was 20.7%. This overall FGID prevalence was similar to a recent Rome Foundation global study, which reported a FGID prevalence of 20.9% amongst persons who completed household surveys in the community [5]. The demographics of our study subjects were representative of the civil service of Malaysia, which represents approximately 15% of the Malaysian population [24]. In particular, the BMI of our study subjects were representative of the average population in Malaysia (71.8% of our study populations vs 64.0% of general populations were either obese or overweight) [25]. In terms of the association between BMI and FGIDs, we observed a trend towards being underweight (i.e. BMI < 18.5) in FGIDs compared to non-FGIDs (6.3% vs 3.5%, P = 0.123) adult subjects, however this did not achieve statistical significance. Amongst the common FGIDs studies, we identified that being underweight (i.e BMI < 18.5) was independently associated with FD, [AOR = 3.648 (95% CI 1.494–8.905), P = 0.004]. However, there was no independent association between BMI and IBS, FC and functional diarrhea.

Our findings are consistent with the results from a large (n = 35,447) population study from France, whereby being underweight was shown to be independently associated with FD amongst females [10]. In contrast, Bouchoucha et al reported that morbid obesity was a risk factor for dyspepsia, but in a tertiary referral centre. A case-control study in another tertiary referral centre, using computerized tomography (CT) scan, demonstrated that visceral adiposity was associated with an increased risk of FD [26]. Amongst a paediatric population (n = 218) in Italy, a positive association was also found between obesity and FD, compared with normal-weight children [27]. In a longitudinal study of 637 subjects in United States, investigators showed that the increase of body weight by >10 lb was associated with dyspepsia-dysmotility over a median of 10.5 years [28]. There may be several reasons for the variation in these observations. Firstly, there were differences in the study population (community, primary care, tertiary care, adult vs children) and the method of measuring weight/ adiposity (BMI vs CT scan). Secondly, differences in BMI criteria are well recognised between Asians and Caucasians [29]. Thirdly, cultural factors in different geographical regions have been shown to influence FGID characteristics and may have contributed to the variation in the association of BMI and FGIDs [30].

The association between a low BMI and FD was not influenced by the presence of anxiety nor depression, when explored in the 2^nd^ phase of the study. FD was independently associated with being underweight (OR 3.231) and with anxiety (OR 2.032). The pathophysiology of FGID is complex, with recognition of gut—brain interaction. Anxiety, but not depression, has been shown to be associated with a referral population [31], as well as increase the risk of developing FD over a 10 year period [32]. Furthermore, anxiety is independently associated with being underweight [33]. Eating disorders, which are often linked with anxiety neurosis, have been reported to be associated with a low BMI and FD at the same time [34,35]. Hence, it may be postulated that FD subjects with a low BMI in our study may have resulted from anxiety and/or eating disorders. Alternatively, dietary restrictions by subjects due to persistent FD symptoms may have led to a low BMI and anxiety. Previous longitudinal studies have suggested that the association between the gut and the brain was bi-directional [36].

This study additionally suggested that functional diarrhea may have been associated with central obesity. This association could not be confirmed by multi-variate analysis due to the small sample size of subjects with functional diarrhea. A recent large population study in the US reported that obesity was positively associated with chronic diarrhea [37]. The population-based study from France mentioned above, had also shown that functional diarrhea was associated with obesity in females [10]. Although BMI associations with IBS have been conflicting, losing weight amongst IBS patients has been shown to improve GI symptoms [38]. Hence it appears that a greater BMI is associated with FGIDs with lower GI symptoms, in contrast to upper GI FGIDs like FD.

There were several limitations in the current study. This study was not a population-based study and it was conducted in a primary care setting involving mainly staff of the hospital and university who were non-GI consulters. However, we were able to reliably rule out significant organic disease (as most subjects had at least basic blood investigations, while those with GI symptoms and warning signs were subjected to endoscopy and/ or imaging as indicated), strengthening the diagnosis of FGID in this study sample. A potential for bias in the diagnosis of FGIDs may have occurred due to the study methodology of using face-to-face interviews. However, as the majority of study subjects were educated, and working in the healthcare environment, there was a lower possibility of GI symptom misinterpretation. The data on psychological disorders (anxiety/ depression) was only obtained from a sub-group of the original subjects in our second phase of study. Nevertheless, the basic demography and prevalence of FGIDs of the sub-group were similar to the original subjects, indicating that they were representative of the original study sample. The sample size of subjects with functional diarrhea was low. However, we were able to demonstrate that functional diarrhea had a positive trend towards association with central obesity. Lastly, the cross-sectional design of our study did not enable us to evaluate the causality between BMI associations with FGID and we did not manage to include dietary habits on the study subjects.

In conclusion, this primary-care based study has demonstrated that being underweight is associated with FD. This association was independent of the presence of anxiety, which was additionally associated with FD. No relation was found between a high BMI and FGIDs. Further studies with a longitudinal design are required to investigate if anxiety causes a low BMI in FD or vice-versa.

## Supporting information

S1 Table Characteristics of study subjects based on individual FGIDs. (DOCX)

S2 Table Basic demographics of study subjects in phase 1 and 2. (DOCX)

S3 Table Univariate and multivariate analysis of risk factors for FGIDs with psychological disorders. (DOCX)

S1 Data (XLSX)
