# Supplementary material for: The association of body mass index with functional dyspepsia is independent of psychological morbidity: A cross-sectional study
Source: PLoS One. 2021 Jan 26;16(1):e0245511. doi: 10.1371/journal.pone.0245511 (PMC7837482; doi:10.1371/journal.pone.0245511)
Supplement: S1 Table — (DOCX) [file pone.0245511.s001.docx]

**S1 Table. Characteristics of Study Subjects based on Individual FGIDs**

|  | **Non-FGID** | **FD** | **IBS** | **Functional Diarrhea** | **FC** |
| --- | --- | --- | --- | --- | --- |
|  | **(N = 795)**  **n (%)** | **(N=75)**  **n (%)** | **(N=40)**  **n (%)** | **(N=12)**  **n (%)** | **(N=105)**  **n (%)** |
| Median Age | 32 (26-40) | 29 (24-41)  P=0.148 | 30 (25-36)  P=0.206 | 28 (24-30)  P=0.019 | 33 (27-38)  P=0.798 |
| Female | 513 (64.5) | 51 (68)  P=0.547 | 24 (60)  P=0.560 | 7 (58.3)  P=0.656 | 76 (72.4)  P=0.112 |
| Body mass index |  |  |  |  |  |
| Less than 18.5 | 28 (3.5) | 10 (13.3)  P=0.002 | 2 (5.0) P=0.698 | 1 (8.3) P=0.219 | 2 (1.9) P=0.320 |
| 18.5 – 22.9 | 191 (24.0) | 17 (22.7)  Reference | 10 (25.0) Reference | 0 | 29 (27.6) Reference |
| 23.0 – 27.4 | 257 (32.3) | 23 (30.7)  P=0.987 | 14 (35.0) P=0.926 | 2 (16.7)  Reference | 36 (34.3). P=0.763 |
| 27.5 and above | 319 (40.1) | 25 (33.3)  P=0.698 | 14 (35.0) P=0.677 | 9 (75.0)  P=0.101 | 38 (36.2) P=0.356 |
| Central obesity | 415 (52.2) | 34 (45.3)  P=0.255 | 20 (50.0)  P=0.786 | 11 (91.7)  P=0.007 | 55 (52.4)  P=0.972 |
| Metabolic Syndrome | 54 (6.8) | 1 (1.3)  P=0.078 | 1 (2,5)  P=0.509 | 0 | 5 (4.8)  P=0.429 |
| Ethnicity  Malay  Chinese  Indian  Others | 723 (90.9)  37 (4.2)  33 (4.7)  2 (0.3) | 65 (86.7)  2 (2.7)  8 (10.7)  0  P=0.069 | 34 (85.0)  1 (2.5)  4 (10.0)  1 (2.5)  P=0.032 | 11 (91.7)  0  1 (8.3)  0  P=0.782 | 98 (93.3)  3 (2.9)  4 (3.8)  0  P=0.796 |
| Education Level  Never schooled  Primary  Secondary  Vocational/college  Tertiary | 2 (0.3)  8 (1.0)  205 (25.8)  420 (52.8)  160 (20.10) | 0  1 (1.3)  20 (26.7)  38 (50.7)  16 (21.3)  P=0.983 | 0  0  8 (20.0)  23 (57.5)  9 (22.5)  P=0.867 | 0  0  1 (8.3)  8 (66.7)  3 (25.0)  P=0.712 | 0  1 (1.0)  33 (31.4)  46 (43.8)  25 (23.8)  P=0.485 |
| Monthly income |  |  |  |  |  |
| Less than USD 750 | 418 (52.6) | 46 (61.3) | 18 (45.0) | 7 (58.3) | 57 (54.3) |
| USD 750 -1249 | 299 (37.6) | 25 (33.3) | 16 (40.0) | 3 (25.0) | 35 (33.3) |
| USD 1250 and above | 78 (9.8) | 4 (5.3) P=0.249 | 6 (15.0) P=0.473 | 2 (16.7) P=0.566 | 13 (12.4) P=0.573 |
| Physical activities  Low  Moderate  High  Unknown | 365 (45.9)  247 (31.1)  107 (13.5)  76 (9.6) | 29 (38.7)  29 (38.7)  11 (14.7)  6 (8.0)  P=0.514 | 14 (35.0)  16 (40.0)  5 (12.5)  5 (12.5)  P=0.507 | 3 (25.0)  4 (33.3)  4 (33.3)  1 (8.3)  P=0.206 | 43 (41.0)  36 (34.3)  16 (15.2)  10 (9.5)  P=0.796 |
| Smoking status  Non smoker  Former smoker  Current smoker | 658 (82.8)  84 (10.6)  53 (6.7) | 62 (82.7)  8 (10.7)  5 (6.7)  P=1.000 | 35 (87.5)  2 (5.0)  3 (7.5)  P=0.525 | 11 (91.7)  1 (8.3)  0  P=0.616 | 87 (82.9)  6 (5.7)  12 (11.4)  P=0.078 |
| Alcohol drinker  Lifetime abstainer  Former drinker  Current drinker | 745 (93.7)  20 (2.5)  30 (3.8) | 73 (97.3)  1 (1.3)  1 (1.3)  P=0.441 | 35 (87.5)  2 (5.0)  3 (7.5)  P=0.303 | 11 (91.7)  0 1 (8.3)  P=0.623 | 100 (95.2)  1 (1.0)  4 (3.8)  P=0.608 |

*FGID, Functional gastrointestinal disorder; FD, Functional dyspepsia; IBS, Irritable bowel syndrome; FC, Functional constipation; USD, US Dollar*
