# Supplementary material for: The association of body mass index with functional dyspepsia is independent of psychological morbidity: A cross-sectional study
Source: PLoS One. 2021 Jan 26;16(1):e0245511. doi: 10.1371/journal.pone.0245511 (PMC7837482; doi:10.1371/journal.pone.0245511)
Supplement: S2 Table — (DOCX) [file pone.0245511.s002.docx]

**S2 Table. Basic demographics of Study Subjects in Phase 1 and 2**

|  | Phase 1  N=1002 | Phase 2  N=694 |
| --- | --- | --- |
| Median age (years) | 32 | 32 |
| Female gender (%) | 65.4 | 67.0 |
| Malay ethnicity (%) | 90.7 | 91.4 |
| Underweight (%) | 4.1 | 4.0 |
| Obesity (%) | 39.2 | 38.2 |
| Central obesity (%) | 51.7 | 50.6 |
| Metabolic syndrome (%) | 6.1 | 5.6 |
| >Secondary level of education (%) | 73.2 | 74.6 |
| Low physical activity (%) | 44.7 | 45.0 |
