# Supplementary material for: The association of body mass index with functional dyspepsia is independent of psychological morbidity: A cross-sectional study
Source: PLoS One. 2021 Jan 26;16(1):e0245511. doi: 10.1371/journal.pone.0245511 (PMC7837482; doi:10.1371/journal.pone.0245511)
Supplement: S3 Table — (DOCX) [file pone.0245511.s003.docx]

**S3 Table. Univariate and multivariate analysis of risk factors for FGIDs with psychological disorders**

|  | **Non-FGID** | **FGID** | | |
| --- | --- | --- | --- | --- |
|  | **N=552** | **N=142** | | |
|  |  |  | **Univariate/ Multivariate analysis** | |
|  | **n (%)** | **n (%)** | **OR (95% CI)** | **AOR (95% CI)** |
| Psychological Disorders |  |  |  |  |
| Anxiety | 117 (21.2) | 42 (29.6) P=0.043 | 1.562 (1.032-2.363) P=0.035 | 1.494 (0.983-2.269) P=0.060 |
| Depression | 73 (13.2) | 23 (16.2) P=0.344 |  |  |
| Median age | 32 (25-41) | 31 (25-38) P=0.129 | 0.985 (0.968-1.003) P=0.095 | 0.993 (0.975-1.011) P=0.421 |
| Female | 364 (65.9) | 101 (78.3) P=0.271 | 1.272 (0.850-1.904) P=0.242 | 1.166 (0.772-1.760) P=0.465 |
| Body mass index |  |  |  |  |
| Less than 18.5 | 19 (3.4) | 9 (6.3) |  |  |
| 18.5 – 22.9 | 129 (23.4) | 33 (23.2) |  |  |
| 23.0 – 27.4 | 190 (34.4) | 49 (34.5) |  |  |
| 27.5 and above | 214 (38.8) | 51 (35.9) P=0.459 |  |  |
| Central obesity | 282 (51.1) | 69 (48.6) P=0.638 |  |  |
| Metabolic syndrome | 36 (6.5) | 3 (2.1) P=0.042 | 0.309 (0.094-1.020) P=0.054 | 0.369 (0.109-1.255) P=0.111 |
| Ethnicity |  |  |  |  |
| Malay | 505 (91.5)) | 129 (90.8) |  |  |
| Chinese | 29 (5.3) | 5 (3.5) |  |  |
| Indian | 16 (2.9) | 7 (4.9) |  |  |
| Others | 2 (0.4) | 1 (0.7) P=0.492 |  |  |
| Educational level |  |  |  |  |
| Never schooled | 0 | 0 |  |  |
| Primary | 5 (0.9) | 1 (0.7) |  |  |
| Secondary | 135 (24.5) | 34 (23.9) |  |  |
| Vocational/ college | 304 (55.1) | 75 (52.8) |  |  |
| Tertiary | 108 (19.6) | 32 (22.5) P=0.882 |  |  |
| Monthly income |  |  |  |  |
| Less than USD 750 | 300 (54.3) | 78 (54.9) |  |  |
| USD 750 - 1249 | 198 (35.9) | 48 (33.8) |  |  |
| USD 1250 or above | 54 (9.8) | 16 (11.3) P=0.823 |  |  |
| Smoking status |  |  |  |  |
| Never smoke | 446 (84.4) | 120 (84.5) |  |  |
| Former smoker | 51 (9.2) | 10 (7.0) |  |  |
| Current smoker | 35 (6.3) | 12 (8.5) P=0.506 |  |  |
| Drinking status |  |  |  |  |
| Lifetime abstainer | 515 (93.3) | 134 (94.4) |  |  |
| Ex-drinker | 16 (2.9) | 2 (1.4) |  |  |
| Current drinker | 21 (3.8) | 6 (4.2) P=0.597 |  |  |
| Level of physical activity |  |  |  |  |
| Low | 250 (45.3) | 62 (43.7) |  |  |
| Moderate | 165 (29.9) | 46 (32.4) |  |  |
| High | 81 (14.7) | 20 (14.1) |  |  |
| Unknown | 56 (10.1) | 14 (9.9) P=0.953 |  |  |

*FGID, Functional gastrointestinal disorder; OR, Odd ratio; CI, Confidence interval; AOR, Adjusted odd ratio*
